# Heart failure risk in patients with atrial fibrillation treated with catheter ablation vs antiarrhythmic drugs

**DOI:** 10.1016/j.hroo.2023.09.009

**Published:** 2023-09-29

**Authors:** Megan Gruber, Maximiliano Iglesias, Rahul Khanna, Dongyu Zhang, Saima Karim

**Affiliations:** ∗ Case Western Reserve University School of Medicine, Cleveland, Ohio; † Franchise Health Economics and Market Access, Johnson & Johnson, Irvine, California; ‡ Medical Device Epidemiology and Real-World Data Sciences, Johnson & Johnson, New Brunswick, New Jersey

**Keywords:** Atrial fibrillation, Heart failure, Catheter ablation, Antiarrhythmic drug, Cardiology

## Abstract

**Background:**

Atrial fibrillation (AF) increases heart failure (HF) risk. Whereas the risk of HF-related hospitalization and mortality are known in the setting of AF, the impact of AF treatment on HF development is understudied.

**Objective:**

The purpose of this study was to compare HF incidence among AF patients treated with antiarrhythmic drugs (AADs) vs catheter ablation (CA).

**Methods:**

AF patients with 1 prior AAD usage were identified in 2014–2022 Optum Clinformatics database. Patients were classified into 2 cohorts: those receiving CA vs those receiving a different AAD prescription. The 2 cohorts were matched on sociodemographic and clinical covariates using propensity score matching technique. Cox regression model was used to compare incident HF risk in the 2 cohorts. Subgroup analyses were performed by race/ethnicity, sex, AF subtype, and CHA_2_DS_2_-VASc score.

**Results:**

After matching, 9246 patients were identified in each cohort (AAD and CA). Patients receiving CA had a 57% lower risk of incident HF than those treated with AADs (hazard ratio [HR] 0.43; 95% confidence interval [CI] 0.40–0.46). Subgroup analysis by race/ethnicity depicted similar results, with non-Hispanic White (HR 0.43; 95% CI 0.40–0.46), non-Hispanic Black (HR 0.46; 95% CI 0.35–0.60), Hispanic (HR 0.53; 95% CI 0.40–0.70), and Asian (HR 0.46; 95% CI 0.24–0.92) patients treated with CA (vs AAD) having significantly lower risk of HF, respectively. The effect size of CA remained significant in subgroups defined by sex, AF subtypes, and CHA_2_DS_2_-VASc score.

**Conclusion:**

AF patients receiving CA had ∼57% lower risk of developing HF than those receiving AAD. The lower risk of HF associated with CA vs AAD persisted across different race/ethnicity, sex, AF subtypes, and CHA2DS2-VASc score.

## Graphical abstract

**Figure:**
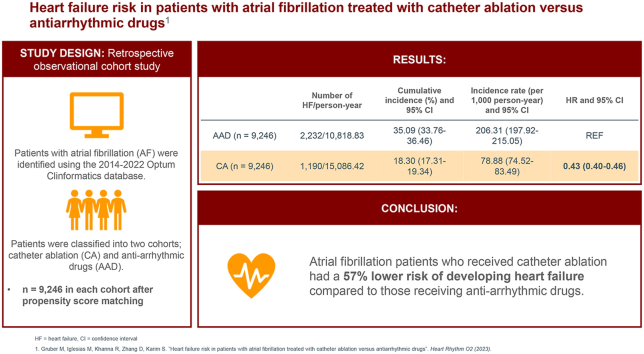

**Key Findings**

▪ In a retrospective analysis of atrial fibrillation (AF) patients with a history of antiarrhythmic drug (AAD) treatment using a national representative administrative claims database, those who underwent a catheter ablation (CA) procedure were observed to have a significantly lower risk of developing heart failure (HF) compared to those who continued AAD treatment.
▪ In this study, the difference in HF risk by treatment modality (CA vs AAD) was consistent across different categories of race/ethnicity, sex, AF subtype, and comorbidity burden.
▪ Based on the results, our study suggests that use of CA vs AAD for AF treatment could alleviate the risk of HF.

## Introduction

Atrial fibrillation (AF) is the most common type of cardiac arrhythmia, with the risk of developing AF being up to 25% for both men and women in their lifetime.^1^ AF affects more than 5.5 million people in the United States (US) and more than 33 million people worldwide.^2^ Epidemiologic evidence suggests that AF is associated with substantial morbidity and mortality.^3^^,^^4^ Studies indicate that individuals with AF have a higher risk of heart failure (HF) compared to individuals without AF, with the 3-year incidence of HF ranging from 2%–20%.^5^, ^6^, ^7^, ^8^, ^9^ HF can cause debilitating symptoms and sequelae, including shortness of breath, peripheral edema, low cardiac output leading to end-organ damage such as pulmonary hypertension, congestive hepatopathy, and renal dysfunction, and can lead to mortaility.^10^^,^^11^ Given the increased risk of HF among individuals with AF, the goal of AF treatment should also be predicated on AF management strategies to mitigate this risk.

Catheter ablation (CA) and antiarrhythmic drugs (AADs) are 2 types of treatment modalities that are widely used to treat patients with AF. Several clinical studies have suggested that CA is a safe and more efficacious alternative to AADs when performed appropriately.^12^, ^13^, ^14^, ^15^ The CABANA (Catheter Ablation Versus Antiarrhythmic Drug Therapy for Atrial Fibrillation) trial is a multicenter, prospective, randomized, open-label clinical trial comparing outcomes among AF patients receiving CA vs pharmacologic treatment.^16^ Although CA (compared to pharmacologic treatment) did not significantly reduce risk of the primary endpoint (death, disabling stroke, serious bleeding, and cardiac arrest) in CABANA,^16^ analysis for secondary outcomes suggested that patients receiving CA had a lower risk of death or cardiovascular hospitalization (hazard ratio [HR] 0.83; 95% confidence interval [CI] 0.74–0.93) and AF recurrence (HR 0.52; 95% CI 0.45–0.60) than those receiving pharmacologic treatment (when analyzed by intent-to-treat approach).^17^ CA has been demonstrated to have superior efficacy with decreased recurrence rates of atrial arrhythmias and reduced progression from paroxysmal to persistent AF compared to AADs in paroxysmal AF patients.^18^, ^19^, ^20^

Although previous studies comparing CA with AAD have provided useful information including variation in HF-related hospitalization and mortality dependent of mode of AF treatment,^21^^,^^22^ the risk of development of new-onset HF in AF patients receiving CA vs AAD has not been studied. Given the established relationship between AF and HF, we hypothesized that a more effective treatment of AF could potentially mitigate the risk of development of HF. Therefore, the purpose of this study was to compare the incidence of development of new-onset HF among cohorts of patients with AF treated with CA vs AAD. In addition, assessment of HF incidence for CA vs AAD by race/ethnicity and sex was performed.

## Methods

### Data source

In this retrospective cohort study, we used data from the Optum Clinformatics Extended Data Mart-Socio-Economic Status (SES) Database between January 2013 and June 2022. The Optum database is a deidentified administrative claims database containing information from commercially insured individuals or individuals with Medicare Advantage health plans. This database has inpatient, outpatient, and pharmacy claims from approximately 18 million covered lives annually.^23^ Furthermore, it contains information on SES (eg, education and income) and location for individuals with both medical and pharmacy coverage at the US Census Division level.^24^ This analysis of the Optum Clinformatics database was conducted under an exemption from Institutional Review Board oversight for US-based studies using de-identified health care records, as dictated by Title 45 Code of Federal Regulations (45 CFR 46.101(b)(4)) (https://www.govinfo.gov/content/pkg/CFR-2011-title45-vol1/pdf/CFR-2011-title45-vol1.pdf). As Optum data do not contain direct identifiers of individuals, employers, households, or providers, Institutional Review Board approval was not required. The research reported in this article adhered to Helsinki Declaration guidelines.

### Study sample

**Figure 1:**
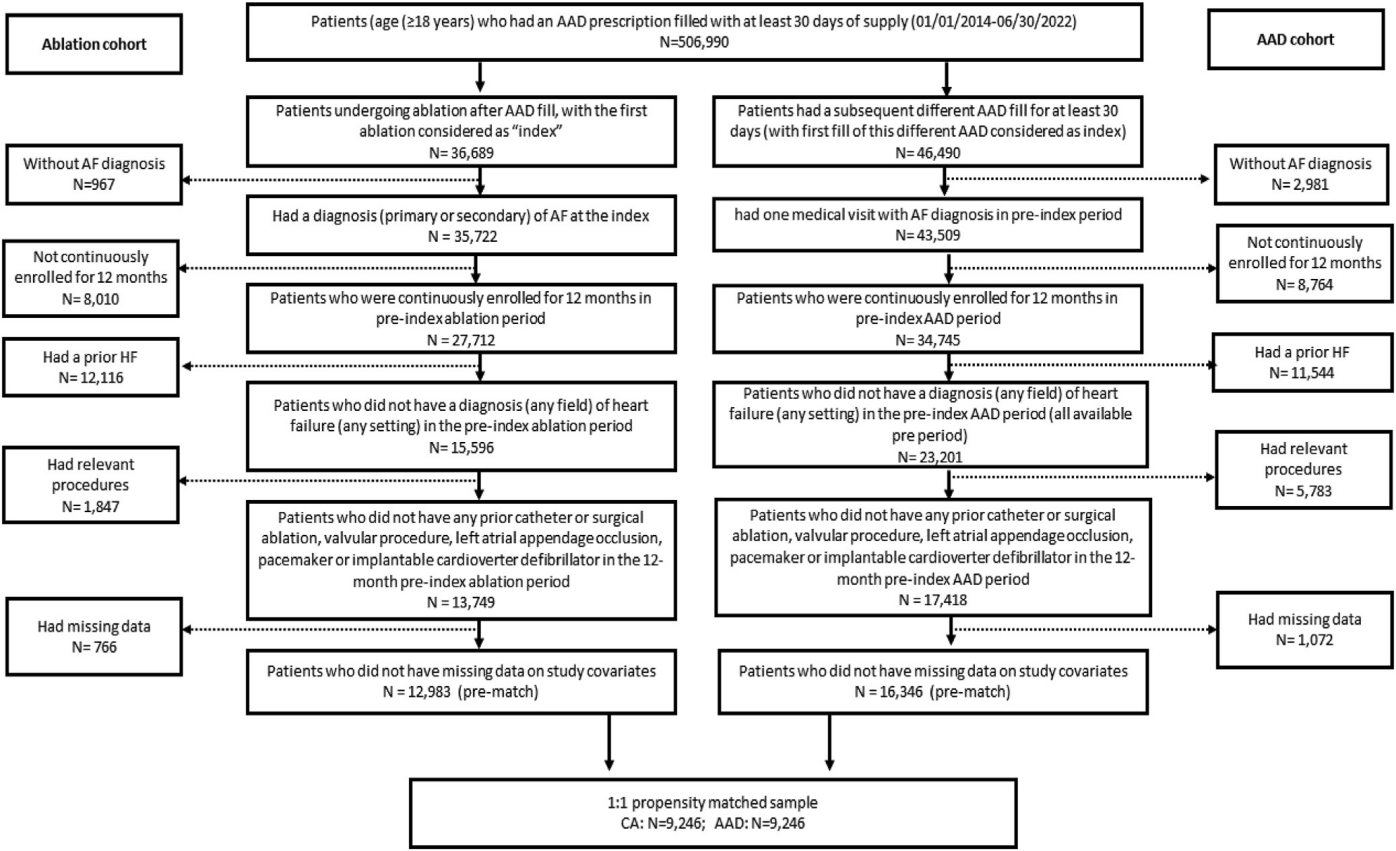
Flowchart of participant selection. AAD = antiarrhythmic drug; AF = atrial fibrillation; CA = catheter ablation; HF = heart failure.

For study purposes, we identified patients (age ≥18 years) with AF and a history of AAD prescription use (amiodarone, dofetilide, dronedarone, flecainide, propafenone, sotalol, disopyramide, quinidine) for ≥30 days between January 01, 2014, and June 30, 2022. These patients with AF were then further categorized into 2 study cohorts: those who received CA vs those who were managed with AAD. Patients included in the CA cohort met the following criteria: (1) received CA (based on Current Procedural Terminology [CPT] 93656 and/or International Classification of Disease, Tenth Revision, Procedure Coding System [ICD-10-PCS] codes 02553ZZ, 02563ZZ, 02573ZZ, 02583ZZ, 025K3ZZ, 025L3ZZ, 025M3ZZ, 025S3ZZ, 025T3ZZ and/or International Classification of Disease, Ninth Revision, Clinical Modification [ICD-9-CM] code 37.34) after the initial AAD prescription fill (with the first CA as the index); (2) had AF diagnosis at the time of index CA; (3) had continuous enrollment for at least 12 months before the index CA; (4) did not have diagnosis of HF in pre-index CA period; (5) did not have any previous catheter or surgical ablation, valvular procedure, left atrial appendage occlusion, pacemaker, or implantable cardioverter-defibrillator in the 12-month preindex CA period; and (6) had no missing data on study covariates. For patients in the AAD cohort, the following criteria were considered for inclusion: (1) had a subsequent fill for at least 30 days for a different AAD after the initial AAD prescription (with first fill date of the second AAD considered as the index date); (2) had at least 1 medical visit with diagnosis of AF in the preindex AAD period; and (3) had the same condition as criteria 3–6 listed for CA cohort. This yielded a total of 12,983 and 16,346 AF patients for the CA and AAD cohorts, respectively. Detailed information regarding cohort selection is given in Figure 1.

### Study outcome

Incident HF was the outcome of interest in our study, and it was assessed based on medical services claim with a diagnosis of HF (ICD-9-CM 39891, 40201, 40211, 40291, 40401, 40403, 40411, 40413, 40491, 40493, 428, 4280, 4281, 4282, 42821, 42822, 42823, 4283, 42831, 42832, 42833, 4284, 42841, 42842, 42843, 4289; ICD-10-CM I099, I110, I130, I132, I50, I501, I502, I5020, I5021, I5022, I5023, I503, I5030, I5031, I5032, I5033, I504, I5040, I5041, I5042, I5043, I50810, I50811, I50812, I50813, I50814, I5082, I5083, I5084, I5089, I509, P290) within 3 years of index treatment date. For patients with multiple HF diagnoses after the index, the first such medical services visit with a diagnosis of HF was treated as outcome of interest for analysis.

### Study covariates

Patient sociodemographics including age, sex, race/ethnicity, region, education, and annual household income were assessed. Sex was categorized as male and female. Race/ethnicity were categorized as non-Hispanic White (NHW), non-Hispanic Black (NHB), Hispanic, Asian, and other. Geographic regions were categorized based on the US Census Bureau regions: Midwest, Northeast, South, and West.^25^ Education was categorized into high school or lower, without a bachelor degree, bachelor or higher; annual household income was categorized into <$50K, $50K–$99.9K, and ≥$100K.^26^ Patient clinical characteristics including Elixhauser Comorbidity Index and CHA_2_DS_2_-VASc were assessed. Insurance type (Medicare, commercial insurance, both) and type of insurance plan (health maintenance organization [HMO] or exclusive provider organization [EPO], preferred provider organization [PPO], point of service [POS] or indemnity, other) were assessed. Utilization of prescription drugs including beta-blockers, calcium channel blockers, and oral anticoagulants were also defined.

### Statistical analysis

Propensity score (PS) matching, with 1:1 ratio and greedy match algorithm with 0.10 caliper, was used to match patients in CA and AAD cohorts on all study covariates. After PS matching, we descriptively summarized study characteristics in both CA and AAD cohorts as well. Standardized mean difference (SMD) was used to assess whether distributions of study covariates were well balanced in the 2 cohorts, and |SMD| <0.25 suggested a balanced distribution.^27^

After matching, we summarized the person-year contributed by study participants, number of incident HF in each cohort, and incidence rate of HF during follow-up. We used Kaplan-Meier curve to visualize risk of incident HF and estimate the cumulative incidence of HF in AF patients. A log-rank test was used to assess whether risk differed by treatment modalities. Specifically, in time-to-event analysis, patients entered the risk set at the index and were followed until the diagnosis of incident HF, disenrollment, end of the 3-year follow-up, or uptake of CA (only for AAD cohort), whichever occurred first. A Cox proportional hazards regression model was used to compare risk of incident HF in postmatching cohorts (CA vs AAD), with AAD cohort as the reference group. HR and 95% CI were used as the measure of association. The proportional hazards assumption of the Cox model was examined by visual inspection of log(−log[S]) plot,^28^ and there was no violation of this assumption.

To explore whether the difference in risk of HF between treatment modalities (CA vs ADD) varied by race/ethnicity (NHW, NHB, Hispanic, and Asian) and sex (female and male), we conducted subgroup analyses for each of these subpopulations. A previous global survey suggested that HF was more common among persistent AF patients than those with paroxysmal AF^2^ ; thus, we performed the analysis in paroxysmal and persistent AF patients, respectively, to explore whether the impact of CA on HF risk varied by AF subtypes. In addition, we conducted a subgroup analysis by CHA_2_DS_2_-VASc score (<4 vs ≥4) because evidence based on the Early Treatment of Atrial Fibrillation for Stroke Prevention Trial-Atrial Fibrillation Network showed that early rhythm control reduced adverse cardiovascular events only for those with CHA_2_DS_2_-VASc score ≥4.^30^ In each set of subgroup analysis, we first applied the same PS matching methods for the subpopulation. We incorporated all covariates used in primary analysis to estimate PS except the factor used for stratification. We then used the Cox proportional hazards regression model to estimate HR and 95% CI for each group.

We conducted 2 sets of sensitivity analysis to explore the stability of the association of CA (compared to AAD) with HF risk. In the first set of sensitivity analysis, patients in the CA cohort were censored when they received a prescription for AAD after the index CA. In the second set of sensitivity analysis, hospitalization with a primary diagnosis of HF was treated as the outcome of interest in time-to-event analysis.

Two-sided *P* <.05 was considered significant. Statistical analyses were conducted using R software Version 4.1.2 (R Foundation for Statistical Computing, Vienna, Austria) and STATA Version 17 (StataCorp, College Station, TX).

## Results

After applying study inclusion and exclusion criteria, 12,983 and 16,346 AF patients were included in the CA and AAD cohorts, respectively (Table 1). In this prematched population, patients in the CA cohort were younger than those in the AAD cohort (64.79 years, SD 9.85 vs 70.07 years, SD 9.92; SMD 0.534). The CA cohort had a lower proportion of female patients than the AAD cohort (37.00% vs 51.03%; SMD 0.285). Significant differences were seen among the CA and AAD prematch cohorts on other study covariates including income, CHA_2_DS_2_-VASc score, insurance plan type, insurance type, utilization of oral anticoagulant, and AF subtype.

**Table 1:** Characteristics of the study population

	Prematch		SMD	Postmatch		SMD
	CA (n = 12,983)	AAD (n = 16,346)		CA (n = 9246)	AAD (n = 9246)	
	n (%)	n (%)		n (%)	n (%)	
Age (y)						
18–49	981 (7.56)	555 (3.40)	0.469	527 (5.7)	489 (5.29)	0.036
50–59	2556 (19.69)	1790 (10.95)		1500 (16.22)	1437 (15.54)	
60–69	4896 (37.71)	4715 (28.84)		3356 (36.3)	3304 (35.73)	
≥70	4550 (35.05)	9286 (56.81)		3863 (41.78)	4016 (43.43)	
Mean ± SD	64.79 ± 9.85	70.07 ± 9.92	0.534	66.20 ± 9.45	67.46 ± 10.30	0.127
Gender						
Female	4804 (37.00)	8341 (51.03)	0.285	3880 (41.96)	3997 (43.23)	0.026
Male	8179 (63.00)	8005 (48.97)		5366 (58.04)	5249 (56.77)	
Race/ethnicity						
NHW	11,045 (85.07)	13,354 (81.70)	0.099	7770 (84.04)	7779 (84.13)	0.011
NHB	750 (5.78)	1161 (7.10)		570 (6.16)	567 (6.13)	
Hispanic	707 (5.45)	1180 (7.22)		550 (5.95)	551 (5.96)	
Asian	226 (1.74)	346 (2.12)		166 (1.8)	172 (1.86)	
Other	255 (1.96)	305 (1.87)		190 (2.05)	177 (1.91)	
Education						
High school or lower	2336 (17.99)	3839 (23.49)	0.172	1801 (19.48)	1847 (19.98)	0.017
Attended collage	7560 (58.23)	9534 (58.33)		5423 (58.65)	5429 (58.72)	
Bachelor degree or higher	3087 (23.78)	2973 (18.19)		2022 (21.87)	1970 (21.31)	
Annual household income ($)						
<50K	2813 (21.67)	5433 (33.24)	0.316	2298 (24.85)	2328 (25.18)	0.018
50K–99.9K	4925 (37.93)	6403 (39.17)		3638 (39.35)	3688 (39.89)	
≥100k	5245 (40.40)	4510 (27.59)		3310 (35.8)	3230 (34.93)	
Geographic region						
Midwest	3076 (23.69)	3487 (21.33)	0.079	2144 (23.19)	2142 (23.17)	0.019
Northeast	1030 (7.93)	1124 (6.88)		723 (7.82)	687 (7.43)	
South	5843 (45.01)	7572 (46.32)		4220 (45.64)	4200 (45.43)	
West	3034 (23.37)	4163 (25.47)		2159 (23.35)	2217 (23.98)	
Elixhauser Comorbidity Index						
0–1	559 (4.31)	513 (3.14)	0.168	360 (3.89)	353 (3.82)	0.017
2–3	3867 (29.79)	3808 (23.3)		2618 (28.31)	2550 (27.58)	
≥4	8557 (65.91)	12,025 (73.57)		6268 (67.79)	6343 (68.6)	
CHA_2_DS_2_-VASc score						
<2	3308 (25.48)	2050 (12.54)	0.334	1868 (20.2)	1711 (18.51)	0.043
≥2	9675 (74.52)	14,296 (87.46)		7378 (79.8)	7535 (81.49)	
Insurance plan type						
EPO/HMO	1695 (13.06)	2259 (13.82)	0.345	1206 (13.04)	1193 (12.9)	0.047
PPO	671 (5.17)	1018 (6.23)		524 (5.67)	540 (5.84)	
POS/indemnity	4558 (35.11)	3295 (20.16)		2740 (29.63)	2555 (27.63)	
Other	6059 (46.67)	9774 (59.79)		4776 (51.65)	4958 (53.62)	
Insurance type						
Commercial	5803 (44.7)	4286 (26.22)	0.394	3509 (37.95)	3307 (35.77)	0.045
Medicare Advantage	7174 (55.26)	12,047 (73.7)		5732 (61.99)	5933 (64.17)	
Both	6 (0.05)	13 (0.08)		5 (0.05)	6 (0.06)	
Comorbidity						
Obesity	4839 (37.27)	4738 (28.99)	0.177	3179 (34.38)	3104 (33.57)	0.017
Valvular disease	5957 (45.88)	6998 (42.81)	0.062	4106 (44.41)	4061 (43.92)	0.010
Peripheral vascular disorders	2553 (19.66)	3803 (23.27)	0.088	1878 (20.31)	1910 (20.66)	0.009
Pulmonary circulation disorders	802 (6.18)	1791 (10.96)	0.171	672 (7.27)	654 (7.07)	0.008
Chronic pulmonary disease	2859 (22.02)	4580 (28.02)	0.139	2179 (23.57)	2212 (23.92)	0.008
Diabetes	2902 (22.35)	4557 (27.88)	0.128	2232 (24.14)	2251 (24.35)	0.005
Hypertension	10,310 (79.41)	13,892 (84.99)	0.146	7527 (81.41)	7584 (82.02)	0.016
Renal failure	1466 (11.29)	3213 (19.66)	0.233	1232 (13.32)	1224 (13.24)	0.003
Cardiomyopathy	1072 (8.26)	1611 (9.86)	0.056	796 (8.61)	811 (8.77)	0.006
Congenital heart disease	693 (5.34)	544 (3.33)	0.099	416 (4.5)	409 (4.42)	0.004
Sleep apnea	4903 (37.76)	4413 (27.00)	0.232	3151 (34.08)	3039 (32.87)	0.026
Smoking	4973 (38.30)	5272 (32.25)	0.127	3332 (36.04)	3317 (35.87)	0.003
Alcohol abuse	389 (3.00)	446 (2.73)	0.016	264 (2.86)	265 (2.87)	0.001
Beta-blocker user	9566 (73.68)	13,165 (80.54)	0.164	7026 (75.99)	7117 (76.97)	0.023
Calcium channel blocker user	2043 (15.74)	3447 (21.09)	0.138	1599 (17.29)	1618 (17.5)	0.005
Oral anticoagulant user	11,288 (86.94)	12,466 (76.26)	0.278	7787 (84.22)	7771 (84.05)	0.005
AF subtype						
Paroxysmal	6467 (49.81)	7796 (47.69)	0.659	4943 (53.46)	5273 (57.03)	0.082
Persistent	4494 (34.61)	2173 (13.29)		2374 (25.68)	2074 (22.43)	
Unspecified	2022 (15.57)	6377 (39.01)		1929 (20.86)	1899 (20.54)	
Interval between initial AAD and index						
<1 y	7692 (59.25)	7760 (47.47)	0.238	5106 (55.22)	4996 (54.03)	0.024
≥1 y	5291 (40.75)	8586 (52.53)		4140 (44.78)	4250 (45.97)	

Absolute values of standardized mean difference (SMD) are reported.

AAD = antiarrhythmic drug; AF = atrial fibrillation; CA = catheter ablation; EPO = exclusive provider organization; HMO = health maintenance organization; NHB = non-Hispanic Black; NHW = non-Hispanic White; POS = point of service; PPO = preferred provider organization.

After PS matching on all study covariates (as listed in the Study covariates section), a total of 18,492 matched patients were identified, with 9246 patients each in the CA and AAD cohorts, respectively. Postmatching, the CA and AAD cohorts were well balanced on all study covariates, as suggested by SMD values (Table 1).

**Figure 2:**
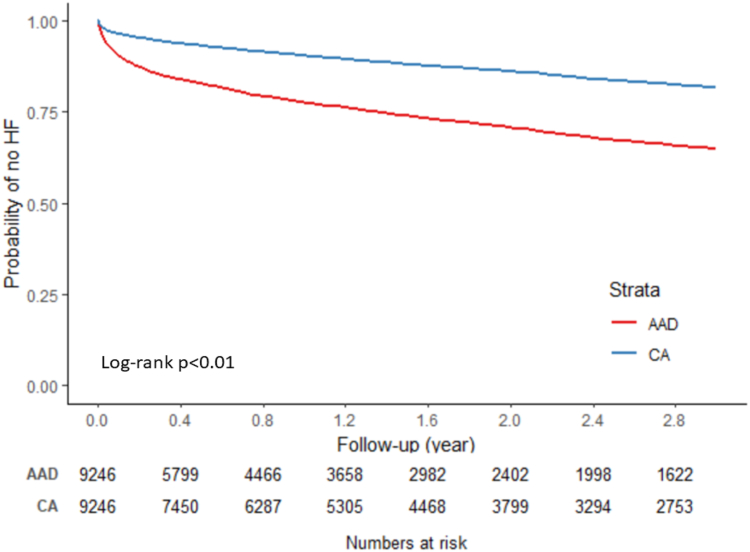
Kaplan-Meier curve of incidence of heart failure (HF) by treatment modality. AAD = antiarrhythmic drug; CA = catheter ablation.

When examining the Kaplan-Meier curves (Figure 2) along with the log-rank test, results suggested that the risk of incident HF was significantly lower in the CA cohort (log-rank P <.01). Overall, median follow-up for the sample was 1.14 years, with 3422 incident HF (CA: 1190; AAD: 2232) cases identified during follow-up (Table 2). The CA cohort had a lower cumulative incidence (CA: 18.30%; AAD: 35.09%) and incidence rate (CA: 78.88; AAD: 206.31 per 1000 person-years) of HF than the AAD cohort. Results from Cox regression model (Table 2) depicted a 57% lower risk of incident HF among patients in the CA cohort compared to those in the AAD cohort (HR 0.43; 95% CI 0.40–0.46).

**Table 2:** Risk of HF by treatment modality

	No. HF/person-year	Cumulative incidence [%] (95% CI)	Incidence rate [per 1000 person-years] (95% CI)	HR (95% CI)
AAD (N = 9246)	2232/10,818.83	35.09 (33.76–36.46)	206.31 (197.92–215.05)	REF
CA (N = 9246)	1190/15,086.42	18.30 (17.31–19.34)	78.88 (74.52–83.49)	0.43 (0.40–0.46)

AAD = antiarrhythmic drug; CA = catheter ablation; CI = confidence interval; HF = heart failure; HR = hazard ratio.

**Figure 3:**
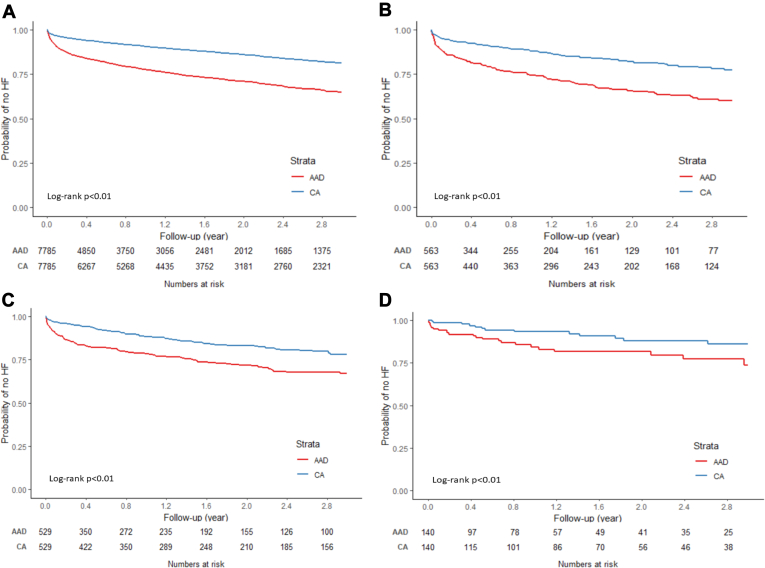
Kaplan-Meier curves of incidence of heart failure (HF) by treatment modality in different race/ethnicity groups: non-Hispanic White **(A)**, non-Hispanic Black **(B)**, Hispanic **(C)**, and Asian **(D)**. AAD = antiarrhythmic drug; CA = catheter ablation.

Similar results were observed when examined by race/ethnicity and sex. When examining Kaplan-Meier curves for NHW, NHB, Hispanic, and Asian patients, the risk of incident HF was observed to be significantly lower for the CA cohort compared to the AAD cohort (log-rank test *P* <.01 for NHW, NHB, Hispanic, and Asian, respectively; Figures 3A–3D). Results from Cox regression analysis showed that CA (vs AAD) was associated with a 57% lower risk of HF among NHW patients (HR 0.43; 95% CI 0.40–0.46). Risk reduction was 54%, 47%, and 54% for NHB (HR 0.46; 95% CI 0.35–0.60), Hispanic (HR 0.53; 95% CI 0.40–0.70), and Asian (HR 0.46; 95% CI 0.24–0.92) patients, respectively (Table 3).

**Table 3:** Risk of HF by treatment modality in subgroups defined by race/ethnicity

	No. HF/person-year	Cumulative incidence [%] (95% CI)	Incidence rate [per 1000 person-years] (95% CI)	HR (95% CI)
NHW				
AAD (N = 7785)	1873/9068.28	35.20 (33.73–36.71)	206.54 (197.40–216.11)	REF
CA (N = 7785)	1002/12,670.04	18.58 (17.49–19.73)	79.08 (74.34–84.14)	0.43 (0.40–0.46)
NHB				
AAD (N = 563)	154/606.57	40.06 (34.50–46.15)	253.89 (216.79–297.32)	REF
CA (N = 563)	87/839.24	22.59 (27.64–18.34)	103.67 (84.02–127.91)	0.46 (0.35–0.60)
Hispanic				
AAD (N = 529)	128/663.98	32.94 (28.03–38.46)	192.78 (162.11–229.24)	REF
CA (N = 529)	80/843.08	21.78 (17.64–26.72)	94.89 (76.22–118.14)	0.53 (0.40–0.70)
Asian				
AAD (N = 140)	23/176.56	26.19 (16.90–39.23)	130.26 (86.56–196.03)	REF
CA (N = 140)	13/231.12	13.98 (8.05–23.68)	56.25 (32.66–96.87)	0.46 (0.24–0.92)

NHB = non-Hispanic Black; NHW = non-Hispanic White; other abbreviations as in Table 2.

**Figure 4:**
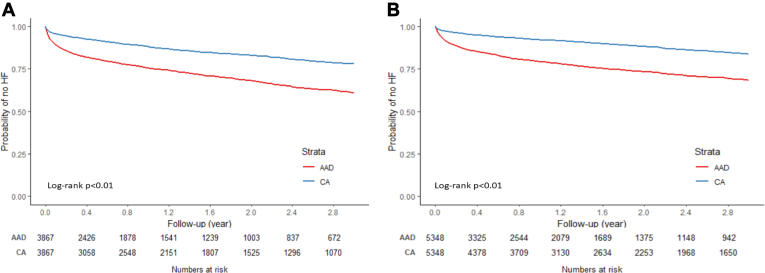
Kaplan-Meier curves of incidence of heart failure (HF) by treatment modality in different sex groups: female **(A)** and male **(B)**. AAD = antiarrhythmic drug; CA = catheter ablation.

When comparing the risk of incident HF among patients treated with CA vs AAD by sex, similar pattern was observed. Among both males and females, a significantly lower risk of incident HF was observed for those treated with CA vs those treated with AAD (Figure 4). For females, the risk of incident HF was 53% lower among those treated with CA vs those treated with AAD; whereas among males, the risk of incident HF was 59% lower for patients treated with CA vs those treated with AAD (Table 4).

**Table 4:** Risk of HF by treatment modality in female and male patients

	No. HF/person-year	Cumulative incidence [%] (95% CI)	Incidence rate [per 1000 person-years] (95% CI)	HR (95% CI)
Female				
AAD (N = 3867)	1053/4519.43	39.02 (36.92–41.19)	232.99 (219.34–247.50)	REF
CA (N = 3867)	605/6113.41	21.99 (20.36–23.72)	98.96 (91.38–107.17)	0.47 (0.43–0.52)
Male				
AAD (N = 5348)	1151/6198.94	31.65 (29.94–33.44)	185.68 (175.25–196.72)	REF
CA (N = 5348)	591/ 8893.62	16.27 (15.02–17.62)	66.45 (61.30–72.03)	0.41 (0.37–0.45)

Abbreviations as in Table 2.

**Figure 5:**
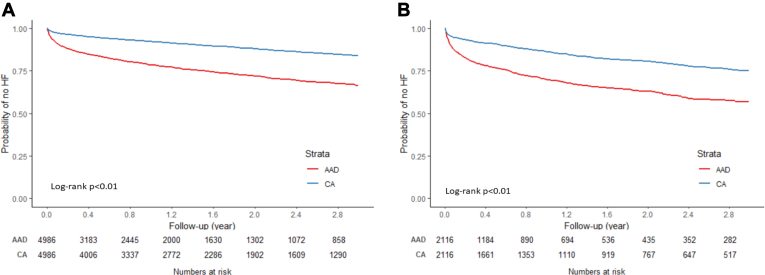
Kaplan-Meier curve of incidence of heart failure (HF) by treatment modality in different atrial fibrillation subtypes: paroxysmal **(A)** and persistent **(B)**. AAD = antiarrhythmic drug; CA = catheter ablation.

**Figure 6:**
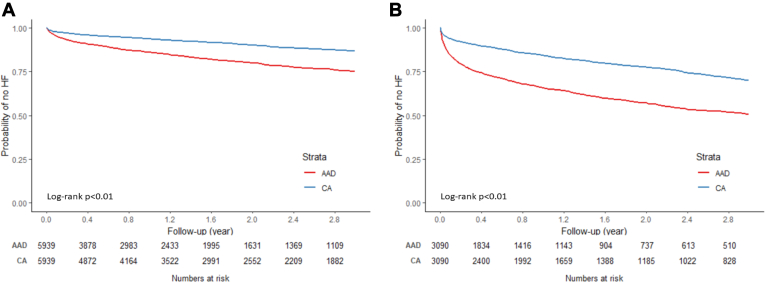
Kaplan-Meier curves of incidence of heart failure (HF) by treatment modality by CHA_2_DS_2_-VASc score <4 **(A)** and score ≥4 **(B)**. AAD = antiarrhythmic drug; CA = catheter ablation.

CA was associated with a lower risk of HF (compared to AAD) in both paroxysmal and persistent AF patients (Figure 5), although the effect size seemed to be slightly larger among paroxysmal AF patients (paroxysmal: HR 0.38, 95% CI 0.34–0.42; persistent: HR 0.45, 95% CI 0.40–0.52) (Table 5). The association patterns of treatment modalities with HF risk were similar in subgroups by CHA_2_DS_2_-VASc score (Figure 6 and Table 6).

**Table 5:** Risk of HF by treatment modality in paroxysmal and persistent atrial fibrillation

	No. HF/person-year	Cumulative incidence [%] (95% CI)	Incidence rate [per 1000 person-years] (95% CI)	HR (95% CI)
Paroxysmal				
AAD (N = 4986)	1148/5888.42	33.55 (31.75–35.42)	194.96 (184.00–206.57)	REF
CA (N = 4986)	528/7836.45	16.12 (14.78–17.56)	67.38 (61.87–73.38)	0.38 (0.34–0.42)
Persistent				
AAD (N = 2116)	637/2114.64	43.02 (40.09–46.08)	301.23 (278.73–325.56)	REF
CA (N = 2116)	372/3180.89	25.01 (22.67–27.54)	116.95 (105.65–129.46)	0.45 (0.40–0.52)

Abbreviations as in Table 2.

**Table 6:** Risk of HF by treatment modality in subgroups by CHA_2_DS_2_-VASc score

	No. HF/person-year	Cumulative incidence [%] (95% CI)	Incidence rate [per 1000 person-years] (95% CI)	HR (95% CI)
CHA_2_DS_2_-VASc score <4				
AAD (N = 5939)	915/7262.18	24.93 (23.37–26.58)	126.00 (118.09–134.43)	REF
CA (N = 5939)	535/10,002.23	13.23 (12.15–14.40)	53.49 (49.14–58.22)	0.47 (0.42–0.52)
CHA_2_DS_2_-VASc score ≥4				
AAD (N = 3090)	1159/3387.57	49.35 (47.06–51.69)	342.13 (322.99–362.41)	REF
CA (N = 3090)	661/4770.55	29.80 (27.78–31.94)	138.56 (128.39–149.53)	0.46 (0.42–0.50)

Abbreviations as in Table 2.

In the first set of sensitivity analysis, 5950 patients (64.35%) in the CA cohort had AAD prescriptions after the index, and 653 patients in the CA cohort who developed HF in the primary analysis were censored due to postindex AAD prescription; however, the association remained inverse and significant (HR 0.39; 95% CI 0.35–0.42) (Supplemental Figure 1 and Supplemental Table 1). The effect measure did not change substantially when we used HF hospitalization as the outcome of interest in the analysis (HR 0.45; 95% CI 0.39–0.53) (Supplemental Figure 2 and Supplemental Table 2).

## Discussion

With previous data establishing the risks of having concomitant HF and AF within an increasingly aging population, it is imperative to afford proper treatment and management of AF. Using a nationally representative large dataset of commercially insured patients in the US, we followed patients with AF to assess the risk of development of HF dependent on the type of rhythm control strategy provided. We included patients who already had received 1 course of AAD therapy, which is consistent with the current HRS/EHRA/ECAS/APHRS/SOLAECE (Heart Rhythm Society/European Heart Rhythm Association/European Cardiac Arrhythmia Society/Asia Pacific Heart Rhythm Society/Sociedad Latinoamericana de Estimulación Cardíaca y Electrofisiología [Latin American Society of Cardiac Stimulation and Electrophysiology]) guidelines that favor ablation when refractory AF or intolerance of antiarrhythmic medications are identified among patients with AF.^28^ Our cohort shows a significant risk of developing new-onset HF in patients with AF, which is consistent with previous global evidence suggesting that HF rates vary between 33% and 56% by AF subtypes.^29^^,^^31^ To the best of our knowledge, this is the first such study to examine and compare the incidence of HF among patients with AF treated with CA vs AAD. In our study, patients with AF receiving CA were observed to have a 57% lower risk of developing incident HF compared to those treated with AAD. Given the novelty of our analysis and the lack of comparable evidence, a direct comparison of our results to previous studies is not feasible; however, a few studies have examined HF-related hospitalizations among patients with AF treated with CA vs AAD. Results from prespecified as-treated analysis from CABANA suggested a 41% reduction in the composite of death or HF hospitalization (HR 0.59; 95% CI 0.41–0.87) for the CA arm vs the AAD arm.^32^ In their analysis of a large retrospective dataset, Jarman et al^22^ observed a 38% lower risk of HF-related hospitalization over a 3-year period for patients with AF treated with CA vs AAD (HR 0.62; *P* = .0318). However, another observational study examining 3-year risk of inpatient admission with HF as the primary diagnosis did not observe any significant difference among patients with AF treated with CA vs AAD (HR_[CA vs AAD]_ = 0.69; 95% CI 0.42–1.15).^21^ Our results, although not directly comparable, suggest that in addition to the potential to reduce the burden of HF, CA treatment could alter the disease course to reduce the risk of incident HF in the first place among AF patients.

The lower risk of incident HF among patients with AF treated with CA vs AAD was observed to be consistent across difference race/ethnicity categories. The effect size of HR in each race/ethnicity group suggests that the potential protective effects of CA in comparison to AAD on incident HF are consistent across NHW, NHB, Hispanic, and Asian patients with AF. The results were also consistent across sex categories, with AF patients treated with CA having significantly lower risk of incident HF than those treated with AAD.

Some factors can explain why AF patients in our ablation cohort had a lower HF risk than counterparts receiving AAD. Among AF patients, the increased heart rate, atrial stretch, and irregularity of the ventricular cycle can contribute to reduced cardiac output, remodeling, and cardiac dysfunction resulting in HF.^33^ Several metaanalyses^34^, ^35^, ^36^ of randomized controlled trials compared outcomes of AF patients receiving CA vs AADs, and the synthesized results suggested that CA was more effective in maintaining sinus rhythm in comparison to antiarrhythmic therapy regardless of medication used. Therefore, by providing improvement of arrhythmia burden and maintaining normal rhythm, CA has better potential to reduce the likelihood of downstream adverse events of AF such as HF.

### Study limitations

This is a retrospective analysis of AF patients who had received either CA or AAD as a second-line therapy. The magnitude of risk of developing HF may not be equivalent to patients who have not received previous antiarrhythmic therapy. This study also excluded patients with surgical or catheter ablation of AF as well as valvular procedures or cardiac implantable device implantation during the 12 months before the index because these procedures could influence incidence of HF. Although we used PS matching to balance the study cohorts on covariates of interest, unmeasured confounding such as severity of AF and left ventricular ejection fraction could have influenced study results. Additionally, misclassification could have occurred during the coding process for the claims and impacted our results. Our study may have misclassification of new-onset HF because the HF diagnosed in outpatient settings could be underreported, and these HF patients might have pre-existing symptoms that were not documented and therefore not new onset. The Optum SES database does not contain information on death, which is an important competing risk of HF during follow-up, making the estimated cumulative incidence biased upward.^37^ The database does not have a measure to reflect postindex AF burden, making us unable to explore whether the lower HF risk is a downstream event of the reduced AF burden. Lastly, the Optum database includes patients with commercial insurance in the US, and our results may not represent uninsured populations.

## Conclusion

In this examination of a large real-world dataset, AF patients with no history of HF who underwent CA were observed to have a significantly lower risk of incident HF compared to those who received AAD. The mitigation of HF risk associated with CA treatment was consistent across different race/ethnicity and sex categories. These results provide decision-making evidence for both clinicians and patients regarding potential subsequent sequelae of AF therapy. A prospective study with measures of AF severity and heart function (eg, left ventricular ejection fraction) will be needed in future to further compare the risk of HF in AF patients treated with CA vs AAD.

## References

[1] Lloyd-Jones D.M., Wang T.J., Leip E.P. (2004). Lifetime risk for development of atrial fibrillation: the Framingham Heart Study. Circulation.

[2] Khavjou O., Phelps D., Leib A. (2016).

[3] Hanley C.M., Esberg D., Kowey P.R. (2014). Ablation versus drugs: what is the best first-line therapy for paroxysmal atrial fibrillation? Antiarrhythmic drugs are outmoded and catheter ablation should be the first-line option for all patients with paroxysmal atrial fibrillation: con. Circ Arrhythm Electrophysiol.

[4] Wu J., Nadarajah R., Nakao Y.M. (2022). Temporal trends and patterns in atrial fibrillation incidence: A population-based study of 3.4 million individuals. Lancet Reg Health Eur.

[5] Andersson T., Magnuson A., Bryngelsson I.L. (2014). Gender-related differences in risk of cardiovascular morbidity and all-cause mortality in patients hospitalized with incident atrial fibrillation without concomitant diseases: a nationwide cohort study of 9519 patients. Int J Cardiol.

[6] Chao T.F., Liu C.J., Tuan T.C. (2018). Lifetime risks, projected numbers, and adverse outcomes in asian patients with atrial fibrillation: a report from the Taiwan Nationwide AF Cohort Study. Chest.

[7] Otterstad J.E., Kirwan B.A., Lubsen J. (2006). Incidence and outcome of atrial fibrillation in stable symptomatic coronary disease. Scand Cardiovasc J.

[8] Ruigomez A., Johansson S., Wallander M.A., Edvardsson N., Garcia Rodriguez L.A. (2009). Risk of cardiovascular and cerebrovascular events after atrial fibrillation diagnosis. Int J Cardiol.

[9] Odutayo A., Wong C.X., Hsiao A.J., Hopewell S., Altman D.G., Emdin C.A. (2016). Atrial fibrillation and risks of cardiovascular disease, renal disease, and death: systematic review and meta-analysis. BMJ.

[10] Groenewegen A., Rutten F.H., Mosterd A., Hoes A.W. (2020). Epidemiology of heart failure. Eur J Heart Fail.

[11] Roger V.L. (2021). Epidemiology of heart failure: a contemporary perspective. Circ Res.

[12] Tardos J.G., Ronk C.J., Patel M.Y., Koren A., Kim M.H. (2021). US antiarrhythmic drug treatment for patients with atrial fibrillation: an insurance claims-based report. J Am Heart Assoc.

[13] Hindricks G., Potpara T., Dagres N. (2021). 2020 ESC Guidelines for the diagnosis and management of atrial fibrillation developed in collaboration with the European Association for Cardio-Thoracic Surgery (EACTS): the Task Force for the diagnosis and management of atrial fibrillation of the European Society of Cardiology (ESC). Developed with the special contribution of the European Heart Rhythm Association (EHRA) of the ESC. Eur Heart J.

[14] Saglietto A., Gaita F., De Ponti R., De Ferrari G.M., Anselmino M. (2021). Catheter ablation vs. anti-arrhythmic drugs as first-line treatment in symptomatic paroxysmal atrial fibrillation: a systematic review and meta-analysis of randomized clinical trials. Front Cardiovasc Med.

[15] Scott L.R. (2016). Antiarrhythmic drugs after ablation for atrial fibrillation: the hope, the hype, and the reality. Eur Heart J.

[16] Chew D.S., Li Y., Cowper P.A. (2022). Cost-effectiveness of Catheter Ablation Versus Antiarrhythmic Drug Therapy in Atrial Fibrillation: the CABANA randomized clinical trial. Circulation.

[17] Packer D.L., Mark D.B., Robb R.A. (2019). Effect of Catheter Ablation vs Antiarrhythmic Drug Therapy on mortality, stroke, bleeding, and cardiac arrest among patients with atrial fibrillation: the CABANA randomized clinical trial. JAMA.

[18] Wazni O.M., Dandamudi G., Sood N. (2021). Cryoballoon ablation as initial therapy for atrial fibrillation. N Engl J Med.

[19] Andrade J.G., Wells G.A., Deyell M.W. (2021). Cryoablation or drug therapy for initial treatment of atrial fibrillation. N Engl J Med.

[20] Kuck K.H., Lebedev D.S., Mikhaylov E.N. (2021). Catheter ablation or medical therapy to delay progression of atrial fibrillation: the randomized controlled atrial fibrillation progression trial (ATTEST). Europace.

[21] Reynolds M.R., Gunnarsson C.L., Hunter T.D. (2012). Health outcomes with catheter ablation or antiarrhythmic drug therapy in atrial fibrillation: results of a propensity-matched analysis. Circ Cardiovasc Qual Outcomes.

[22] Jarman J.W.E., Hussain W., Wong T. (2018). Resource use and clinical outcomes in patients with atrial fibrillation with ablation versus antiarrhythmic drug treatment. BMC Cardiovasc Disord.

[23] Freeman L., Kee A., Tian M., Mehta R. (2021). Retrospective claims analysis of treatment patterns, relapse, utilization, and cost among patients with multiple sclerosis initiating second-line disease-modifying therapy. Drugs Real World Outcomes.

[24] Field M.E., Goldstein L., Corriveau K., Khanna R., Fan X., Gold M.R. (2021). Same-day discharge after catheter ablation in patients with atrial fibrillation in a large nationwide administrative claims database. J Cardiovasc Electrophysiol.

[25] O'Byrne M.L., DeCost G., Katcoff H. (2020). Resource utilization in the first 2 years following operative correction for tetralogy of fallot: study using data from the Optum's De-Identified Clinformatics Data Mart Insurance Claims Database. J Am Heart Assoc.

[26] Zhang Y., Amin S., Lung K.I., Seabury S., Rao N., Toy B.C. (2020). Incidence, prevalence, and risk factors of infectious uveitis and scleritis in the United States: a claims-based analysis. PLoS One.

[27] Stuart E.A., Lee B.K., Leacy F.P. (2013). Prognostic score-based balance measures can be a useful diagnostic for propensity score methods in comparative effectiveness research. J Clin Epidemiol.

[28] Kuitunen I., Ponkilainen V.T., Uimonen M.M., Eskelinen A., Reito A. (2021). Testing the proportional hazards assumption in cox regression and dealing with possible non-proportionality in total joint arthroplasty research: methodological perspectives and review. BMC Musculoskelet Disord.

[29] Chiang C.E., Naditch-Brule L., Murin J. (2012). Distribution and risk profile of paroxysmal, persistent, and permanent atrial fibrillation in routine clinical practice: insight from the real-life global survey evaluating patients with atrial fibrillation international registry. Circ Arrhythm Electrophysiol.

[30] Rillig A., Borof K., Breithardt G. (2022). Early rhythm control in patients with atrial fibrillation and high comorbidity burden. Circulation.

[31] Kotecha D., Piccini J.P. (2015). Atrial fibrillation in heart failure: what should we do?. Eur Heart J.

[32] Packer D.L., Piccini J.P., Monahan K.H. (2021). Ablation versus drug therapy for atrial fibrillation in heart failure: results from the CABANA trial. Circulation.

[33] Anter E., Jessup M., Callans D.J. (2009). Atrial fibrillation and heart failure: treatment considerations for a dual epidemic. Circulation.

[34] Khan A.R., Khan S., Sheikh M.A., Khuder S., Grubb B., Moukarbel G.V. (2014). Catheter ablation and antiarrhythmic drug therapy as first- or second-line therapy in the management of atrial fibrillation: systematic review and meta-analysis. Circ Arrhythm Electrophysiol.

[35] Hakalahti A., Biancari F., Nielsen J.C., Raatikainen M.J. (2015). Radiofrequency ablation vs. antiarrhythmic drug therapy as first line treatment of symptomatic atrial fibrillation: systematic review and meta-analysis. Europace.

[36] Turagam M.K., Musikantow D., Whang W. (2021). Assessment of catheter ablation or antiarrhythmic drugs for first-line therapy of atrial fibrillation: a meta-analysis of randomized clinical trials. JAMA Cardiol.

[37] Abdel-Qadir H., Fang J., Lee D.S. (2018). Importance of considering competing risks in time-to-event analyses: application to stroke risk in a retrospective cohort study of elderly patients with atrial fibrillation. Circ Cardiovasc Qual Outcomes.

