# Supplementary material for: Heart failure risk in patients with atrial fibrillation treated with catheter ablation vs antiarrhythmic drugs
Source: Heart Rhythm O2. 2023 Sep 29;4(11):681–91. doi: 10.1016/j.hroo.2023.09.009 (PMC10685151; doi:10.1016/j.hroo.2023.09.009)
Supplement: Supplementary Data [file mmc1.docx]

**Supplementary Fig 1.** Kaplan-Meier curve for AF patients. In this Kaplan-Meier figure, patients using AAD in ablation cohort were censored. Abbreviations: AAD: antiarrhythmic drug, CA: catheter ablation, HF: heart failure


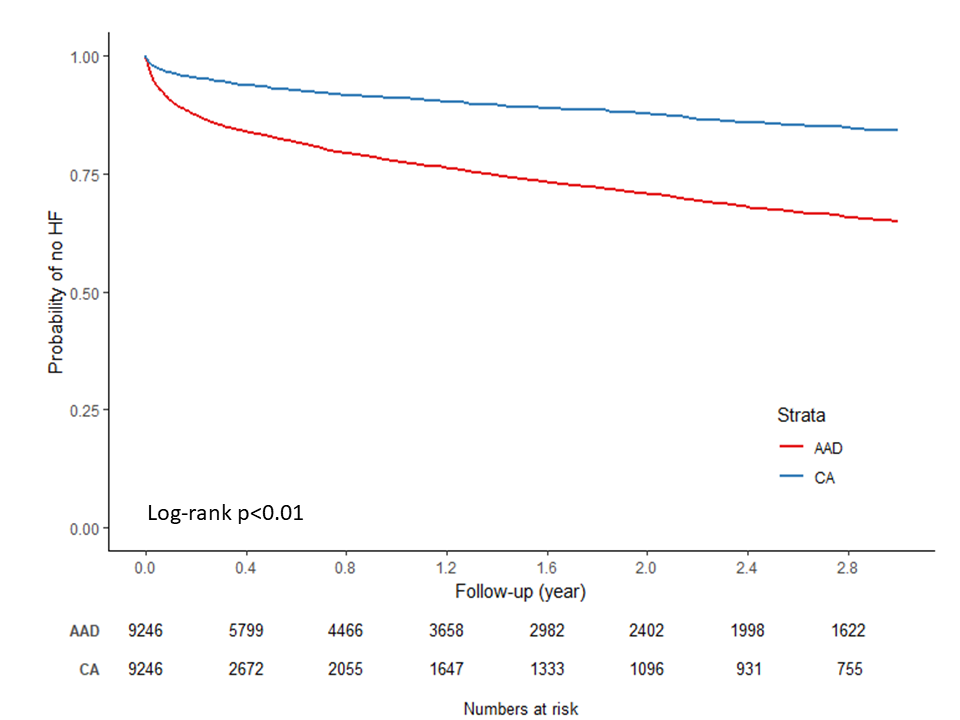


**Supplementary Table 1.** Risk of HF by treatment modalities

|  | **No. HF/Person-year** | **Cumulative incidence (%) and 95% CI** | **Incidence rate (per 1,000 person-year) and 95% CI** | **HR and 95% CI** |
| --- | --- | --- | --- | --- |
| AAD (N=9,246) | 2,232/10,818.83 | 35.09 (33.76, 36.46) | 206.31 (197.92, 215.05) | REF |
| CA (N=9,246) | 537/5,302.73 | 15.93 (14.36, 17.66) | 101.27 (93.06, 110.21) | 0.39 (0.35, 0.42) |

Abbreviations: AAD: antiarrhythmic drug, CA: catheter ablation, CI: confidence interval, HF: heart failure, HR: hazard ratio.

Patients using AAD in ablation arm were censored in this analysis.

**Supplementary Fig 2.** Kaplan-Meier curve for AF patients. In this Kaplan-Meier figure, HF hospitalization was treated as the outcome. Abbreviations: AAD: antiarrhythmic drug, CA: catheter ablation, HF: heart failure


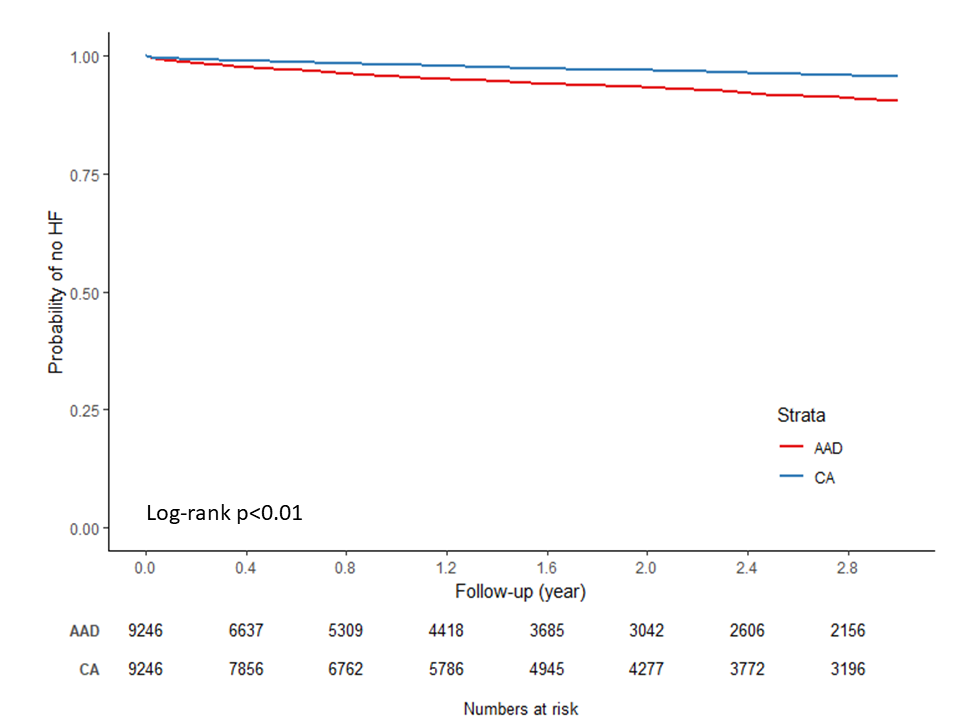


**Supplementary Table 2.** Risk of HF by treatment modalities

|  | **No. HF/Person-year** | **Cumulative incidence (%) and 95% CI** | **Incidence rate (per 1,000 person-year) and 95% CI** | **HR and 95% CI** |
| --- | --- | --- | --- | --- |
| AAD (N=9,246) | 493/12,909.18 | 9.46 (8.62, 10.39) | 38.19 (34.96, 41.71) | REF |
| CA (N=9,246) | 271/16,421.00 | 4.40 (3.89, 4.98) | 16.50 (14.65, 18.59) | 0.45 (0.39, 0.53) |

Abbreviations: AAD: antiarrhythmic drug, CA: catheter ablation, CI: confidence interval, HF: heart failure, HR: hazard ratio.

HF hospitalization was treated as the outcome of interest in this analysis.
